# Prenatal ethanol exposure leads to persistent anxiety-like behavior during adulthood indicated by reduced horizontal and vertical exploratory behaviors

**DOI:** 10.3389/fnins.2023.1163575

**Published:** 2023-04-06

**Authors:** An-Li Wang, Veronika B. Micov, Francis Kwarteng, Ruixiang Wang, Kathryn A. Hausknecht, Saida Oubraim, Samir Haj-Dahmane, Roh-Yu Shen

**Affiliations:** Department of Pharmacology and Toxicology, Jacob School of Medicine and Biomedical Sciences, University at Buffalo, Buffalo, NY, United States

**Keywords:** fetal alcohol spectrum disorders, anxiety-like behavior, open field, acute restraint stress, exploratory rearing

## Abstract

**Background:**

Fetal alcohol spectrum disorders (FASD) caused by prenatal ethanol exposure (PE) consist of many cognitive/behavioral deficits. Studies have reported that PE leads to impairments of learning and memory, attention, executive function, and anxiety. Open field (OF) is a common behavioral model which offers comprehensive ethological information. Here, we analyzed multiple parameters of OF to examine anxiety behavior and habituation after PE.

**Material and Methods:**

Pregnant Sprague Dawley rats were gavaged twice/day with 0 or 3 g/kg/treatment ethanol (15% w/v) during gestational day (GD) 8–20, mimicking second-trimester heavy PE in humans. The control and PE adult offspring were subjected to OF task in different ambient light levels with or without acute stress.

**Results:**

Prenatal ethanol exposure did not influence the overall locomotor activities or habituation in the OF. In lower ambient light, no PE effects could be detected. In higher ambient light, female PE rats showed less activities in the center zone, indicative of increased anxiety. Males show lower activities in the center zone only after acute stress. Rats spent <2% of the time in the center zone compared to >75% of the time in the corner zone where they engaged in frequent rearing activities (vertical exploration; exploratory rearing). Prenatal ethanol exposure led to lower rearing activities in the corner in both males and females. Acute stress masks the PE effects in males but not in females.

**Discussion:**

The results support that heavy PE leads to persistent anxiety-like behavior during adulthood in both sexes. This conclusion is supported by using multiple parameters of exploratory behavior in the OF, including the rearing activities in the corner to reach reliable quantification of anxiety-like behavior.

## Introduction

Prenatal ethanol exposure (PE) leads to structural and functional abnormalities (Tang et al., 2019) in the brain, which can cause lifelong cognitive/behavioral deficits and mood disorders (Pyman et al., 2021). The term fetal alcohol spectrum disorders (FASD) is used to represent multiple cognitive/behavioral/emotional deficits caused by PE. The global prevalence of FASD in children and youth population was estimated to be 7.7 per 1,000 population (Lange et al., 2017). The prevalence rate of FASD is persistently high (2–5%) in the general population of the US (May et al., 2009, 2014). Currently, understanding of FASD pathology and treatment strategies is limited. With such high prevalence and accompanying huge economic costs (Greenmyer et al., 2018), it is crucial to understand the mechanisms caused by PE in order to develop effective intervention strategies.

The cognitive/behavioral deficits in FASD include poor executive function (Kingdon et al., 2016), learning and memory deficits (Mattson et al., 2011), hyperactivity (Mattson et al., 2011) and attention deficits (Pyman et al., 2021). In addition, individuals with FASD also show mood disorders such as anxiety and depression (Famy et al., 1998; Fryer et al., 2007; Hellemans et al., 2010a) and vulnerability to stress (Hellemans et al., 2010a; Keiver et al., 2015). The above deficits can be observed in rodent models of PE including the attention deficit (Wang et al., 2020, 2021), sensory processing deficit, and habituation deficit (Wang et al., 2022). Prenatal ethanol exposure in rodents also leads to depressive-like and anxiety-like behaviors (Hellemans et al., 2010b). The anxiety-like behavior is shown using various behavioral tasks including elevated plus-maze task (Osborn et al., 1998; Dursun et al., 2006; Hwang and Hashimoto-Torii, 2022; Oubraim et al., 2022) and elevated zero maze task (Oubraim et al., 2022), novelty-induced hypophagia, light–dark box, and open field (OF) (Rouzer et al., 2017). In addition, PE reduces social behaviors in the social interaction task (Diaz et al., 2016, 2020). These studies show inconsistent results possibly due to PE paradigms (route, timing, and duration), rodent species/strains, sexes and behavioral tasks used. The elevated plus-maze is one of the most popular ethological models to measure anxiety levels, which has numerous advantages (e.g., does not require training, or water/food deprivation, and simplicity of design) (Carobrez and Bertoglio, 2005). However, the elevated plus-maze task and other similar tasks (e.g., light–dark box) cannot offer comprehensive information related to exploratory behaviors which is important for inferring anxiety.

Open field task, on the other hand, allows us to fully examine exploratory behavior. In the OF task, animals are allowed to explore the entire arena freely. They usually locomote around the walls and avoid the center open area. The behavioral pattern is termed thigmotaxis. Reduced center zone activities represent lower horizontal exploratory activities and higher anxiety levels. In addition to the center zone activities as an index of anxiety, some evidence shows that vertical exploratory behavior (rearing; high-leaning behavior) could also infer anxiety levels in rodents (Kuniishi et al., 2017; Sturman et al., 2018). However, most investigators only use center zone exploratory activities in the OF, ignoring the vertical exploratory behavior (rearing) which takes place much more frequently than center zone activities. So far, there are no studies investigating chronic heavy PE on anxiety-like behavior in both sexes using the OF with detailed analysis of exploratory behavior. In the present study, we investigate the effects of heavy PE during the second-trimester equivalent using detailed analyses of both horizontal and vertical exploratory behaviors. Additionally, we analyzed the anxiety-like behavior after 2 h acute restraint stress (ARS) since animals were more vulnerable to stress after exposure to ethanol prenatally (Hellemans et al., 2010b; Wieczorek et al., 2015).

## Materials and methods

### Animals

Rats were bred in house to prevent potential stress from transportation and to strictly control the prenatal environment. The procedure of breeding, ethanol exposure, and cross-fostering were described in previous reports (Wang et al., 2020, 2021). Briefly, male and virgin female Sprague–Dawley rats (Envigo, Indianapolis, IN, United States) were housed in the breeding cages with food and water *ad lib.* Gestational day 0 is designated when copulatory vaginal plugs were found. The holding room was maintained a 12 h/12 h regular light–dark cycle (lights on 7:00 a.m.) with temperature and humidity well-controlled.

Pregnant dams were randomly assigned to vehicle control (22.5% w/v sucrose water, isocaloric to ethanol) or ethanol (3 g/kg; 15% w/v) group. Our previous study reported that the blood alcohol concentration of this PE paradigm was 116.8 ± 10.5 mg/dl 1 h after the second ethanol treatment on GD 15 (Shen et al., 1999; Wang et al., 2019). This ethanol dosage in rats is comparable to heavy prenatal alcohol exposure in humans (Wang et al., 2020). From GD 8 to GD 20, both groups were administered twice (0 or 3 g/kg ethanol; 5–6 h apart) *via* intragastric gavage every weekday. On weekends, a single treatment with 0 or 4 g/kg ethanol solution was given daily. Control dams were pair-fed with ethanol treated dams on GDs 8–20 to equate nutrient intake from rat chow across groups. Food was *ad lib* after GD 20. Both control and ethanol groups were injected thiamine-containing vitamin B-complex (containing 8 mg/kg; i.m.; twice/week during GDs 8–20; Super B Complex, Vedco, Saint Joseph, MO) to compensate for potential thiamine deficiency caused by ethanol treatment or the pair-feeding condition (Ba et al., 1996).

Each litter was culled to 10 pups with equal number of pups in each sex (5 males and 5 females), when possible, on postnatal day 1. The pups of PE dams were fostered by extra dams without any treatment and gave birth 1–2 day(s) earlier. The cross-fostering procedure was to prevent the potential maternal negligence or undernutrition from alcohol withdrawal of PE dams. The pups of each control dam were switched. However, cross-fostering might lead to other physiological changes and might not be translational to clinical studies (Giberson and Weinberg, 1997; Bartolomucci et al., 2004). Twenty-five male rats (control: 12 from 8 litters; PE: 13 from 8 litters) and 26 female rats (control: 14 from 8 litters; PE: 12 from 8 litters) were utilized in the current study. Before the behavioral experiment start, animals were handled for 3 consecutive days (5 min/rat/day). Animals entered behavioral experiments at 8–9 weeks old. All procedures were followed by the guidelines of the National Institutes of Health regarding laboratory animal care and use, and approved by the Institutional Animal Care and Use Committee of University at Buffalo.

### Apparatus

#### Open field

**Figure 1:**
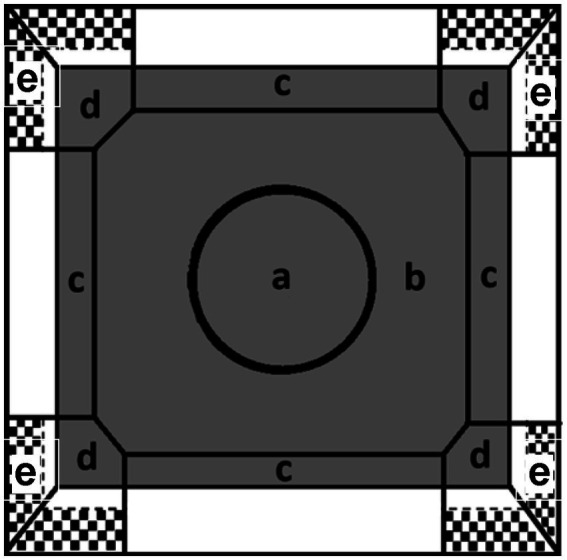
The illustration of each zone in the OF task. (a) Center zone, (b) peripheral zone, (c) edge zone, and (d) corner zone. An additional rearing zone was defined on the upper wall in the corner (e).

Four identical OF apparatuses were used for testing. Each OF apparatus consisted of a square arena (100 × 100 cm) with black rubber floor and 46 cm high walls. The lights offered 2 levels of even illumination: the lower light level (~14 lux at center, ~9 lux at corners of the OF) and the higher light level (~60 lux at center, ~40 lux at corners). White noise was provided in the testing room. One camera was placed 2.4 m above each OF apparatus. All tests were recorded and analyzed automatically with ANY-maze version 6 software (Stoelting Co., Illinois, United States). Each OF was virtually divided into the following zones on the floor (Figure 1): (A) center (at the center area with a diameter of 43 cm), (B) peripheral zone (the area between center and edge zones), (C) edge zone (10 cm distance from each wall), (D) corner zone. The position of the animal in zones A–D was measured with a center point on the torso area. The following variables were collected from zones A–D: distance moved (m), duration (s), frequency (number of entries), and latency(s) of the first-time entry. Area under the curve (AUC) of total distance was calculated to reflect the habituation of the locomotor activities in the OF. Lower AUC represents faster habituation (Wang et al., 2018a). The rearing behavior was measured when animal’s nose position was tracked and went over the virtual line on the wall which was 13 cm above the floor in the corner area (zone E, Figure 1). During rearing, the distance of head movement and head turn angle which are features of exploratory behavior (Lever et al., 2006) were also recorded. For example, rats moved their heads 15 degrees to the left and then moved their heads 45 degrees to the right in a rearing event, the head turn angle would be 60 degrees. In addition, duration (s), frequency (number of entries), head distance moved (m), and latency (s) of the first-time entry of rearing behavior were also recorded.

#### Acute restraint stress

The ARS procedure was applied to enhance psychological stress and without causing physical injury (Briski and Gillen, 2001). Transparent cylinders with ventilation holes were used for ARS. The cylinders were 23 cm long, 6.3 cm in diameter (Model 81, IITC Life Science Inc., United States).

### Behavioral testing

Under the lower ambient light condition, animals were habituated in the OF testing room for 15 min before the OF tests. Each animal was placed in the center of the OF at the start of the test. Each animal was allowed to explore the entire OF freely for 18 min. Four weeks later when animals were 12–13 weeks old, they were habituated in the testing room for 15 min prior to the OF tests under the higher ambient light for 18 min (Baseline). Three weeks later when animals were 15–16 weeks old, an acute restraint stress (ARS) was performed right before the OF test. Under bright light, animals were restrained in the rodent restrainers for 2 h. After the ARS, animals were returned to their home cages and immediately brought to the testing room for 15 min habituation, where animals were allowed to move freely to avoid non-specific motor effects due to movement restriction during restraint. Then the animals were placed in the OF apparatuses for the 18 min test (after ARS).

### Statistics

A two- or three-way repeated measures ANOVA with litter as a nested factor was used in statistical analysis when appropriate. We use statistical approach to control for possible litter effects and increase the rigor of the current study. “Litters” were nested under prenatal treatment conditions and used as a nested factor. Significant litter effects were also reported in statistical analysis. This approach was used successfully for controlling litter effects in our previous studies (Wang et al., 2018b; Aghaie et al., 2020). Lazic and Essioux (2013) argue that the nested design can avoid statistical bias when the whole litter is subject to the same experimental procedure *in utero*. Planned comparison was used after ANOVA for pairwise comparison. Independent *t*-test was applied for the data under lower ambient light condition. Values represented Mean ± SEM. The significant levels were set as *p* ≤ 0.05. All calculations were performed with STATISTICA 7 (Stat Soft. Inc., Oklahoma, United States).

## Results

### Prenatal ethanol exposure slightly lowered litter size and pup weight on postnatal day 1

Eight control and 8 PE dams gave birth to a total 174 pups: 89 males and 85 females, respectively (Table 1). Prenatal treatment decreased the litter size (independent *t*-test: df = 14, *t* = 3.1, *p* < 0.01). Prenatal treatment also slightly decreased the pup weight on postnatal day 1 (two-way repeated measures ANOVA with litter as a nested factor: prenatal treatment, sex: main effect of prenatal treatment, *F*(1, 142) = 20.08, *p* < 0.001, main effect of sex, *F*(1, 142) = 18.21, *p* < 0.001; litter effect, *F*(28, 142) = 4.92, *p* < 0.001). Planned comparison after ANOVA showed prenatal treatment led to slightly lower weight in both male and female pups. These results are consistent with our previous report (Wang et al., 2020), which suggested that the current PE condition did not cause major teratogenic effects.

**Table 1:** Birth outcome.

	Control: 8 L	PE: 8 L	Value of *p*
	(Mean ± SEM)	(Mean ± SEM)	
Litter size	12.50 ± 0.78	9.37 ± 0.65	<0.01
Pup weight on postnatal day 1			
Male pups’ weight (g)	6.33 ± 0.13	5.97 ± 0.12	<0.01
Female pups’ weight (g)	6.02 ± 0.10	5.53 ± 0.13	<0.01

Data are represented as Mean ± SEM. PE, prenatal ethanol exposure.

### Prenatal ethanol exposure did not result in differences in overall locomotor activities in male and female rats under lower ambient light

**Figure 2:**
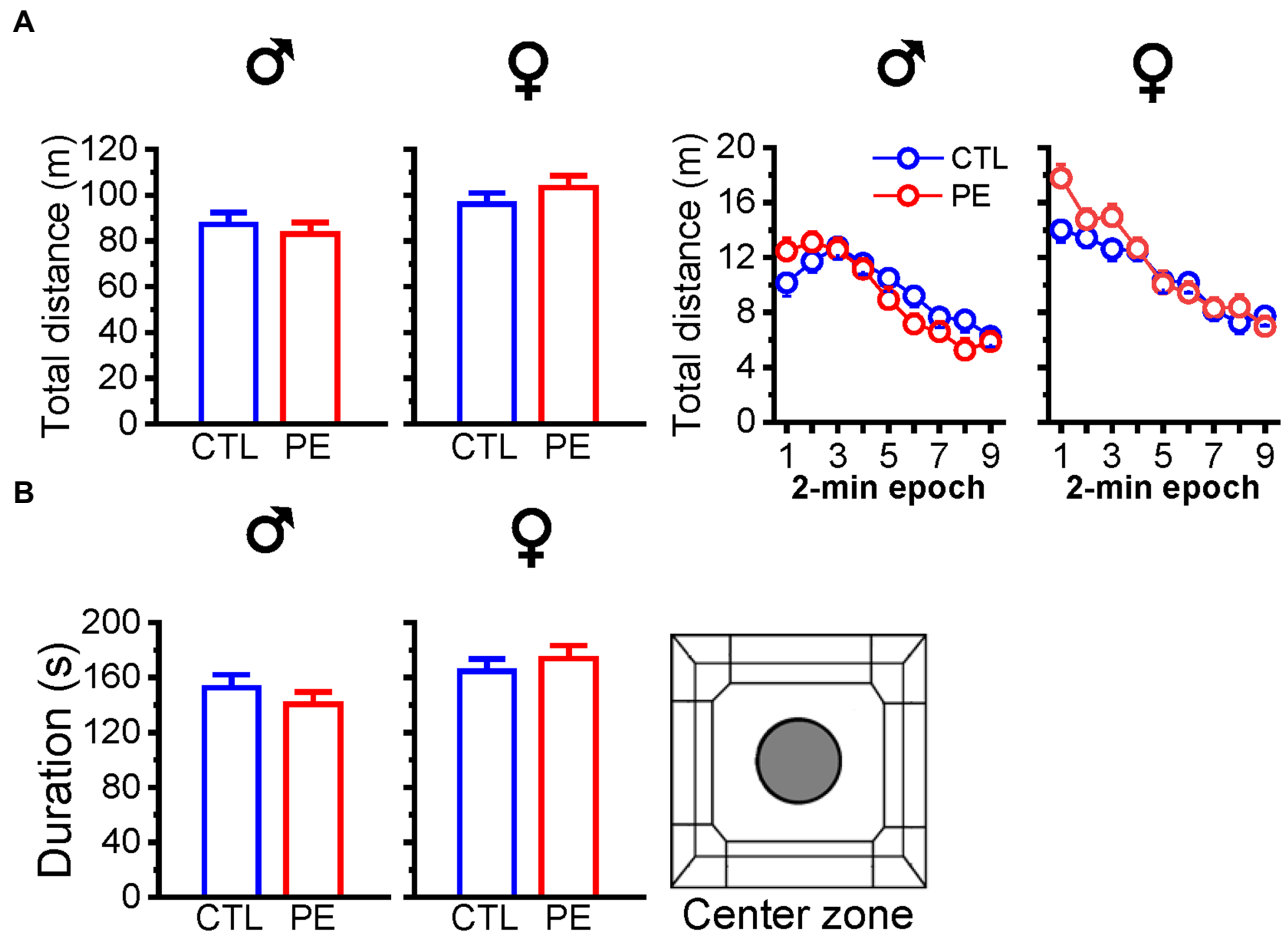
Effects of prenatal ethanol exposure (PE) on total distance in the OF and duration in the center zone in male and female rats under lower ambient light (14 lux). Prenatal ethanol exposure did not alter total distance travelled in the OF **(A)** or duration spent in the center zone **(B)**.

We analyzed the total distance of locomotor activities in the entire OF under lower ambient light. Prenatal ethanol exposure did not impact the total distance in males or females (two-way repeated measures ANOVA with litter as a nested factor: prenatal treatment, 2-min epoch; Figure 2A). In addition, PE did not affect duration in the center zone in either male or female rats (independent *t*-test). The results did not show PE effects in males and females (Figure 2B). When we compare the total distance of locomotor activities under the lower and higher ambient light level under the baseline condition, we did not observe PE effects.

### Prenatal ethanol exposure did not result in differences in overall locomotor activities or habituation in male and female rats under higher ambient light condition

We compared the total distance travelled in the OF under lower and higher ambient light levels and found rats travelled greater distance under lower light condition. In addition, female rats travelled greater distance (three-way repeated measures ANOVA with litter as a nested factor: prenatal treatment, sex, light level: main effect of sex, *F*(1, 23) = 9.06, *p* < 0.01; main effect of light level, *F*(1, 23) = 39.71, *p* < 0.001).

Next, we compare the duration rats spent in the center zone in the OF under lower and higher ambient light levels. We find rats spent significantly less time (<10%) in the center zone under higher ambient light in both sexes (two-way repeated measures ANOVA with litter as a nested factor: sex, light level: interaction effect of sex and light level, *F*(1, 23) = 14.19, *p* < 0.01, planned comparison, male: lower vs. higher light, *p* < 0.001, female: lower vs. higher light, *p* < 0.001).

**Figure 3:**
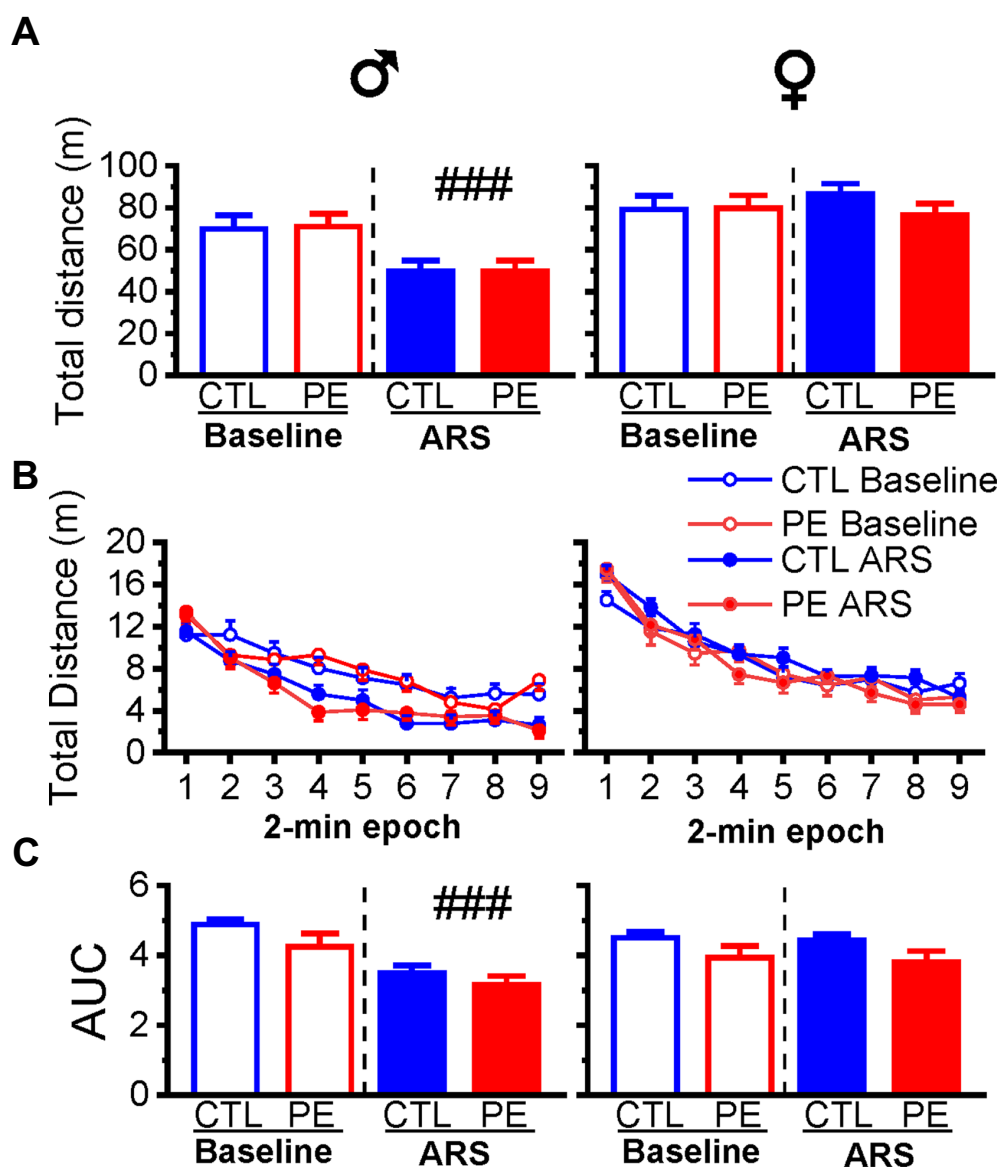
Effects of prenatal ethanol exposure (PE) on locomotor activity and its habituation under higher ambient light (60 lux). Prenatal ethanol exposure did not impact total distance travelled under the basal condition **(A,B)**. In males, acute restraint stress (ARS) decreased total distance travelled in the OF. Prenatal ethanol exposure did not alter habituation of the locomotor activity in the OF in either sex **(C)**. Acute restraint stress facilitated the habituation of locomotor activity shown as reduced value of area under the curve (AUC). Data are represented as Mean ± SEM. ###*p* < 0.001, baseline condition vs. ARS. CTL, control.

In males, we observed ARS reduced distance travelled without any PE effects (three-way repeated measures ANOVA: prenatal treatment, ARS, 2-min epoch with litter as a nested factor: interaction effect of ARS and epoch, *F*(8, 184) = 4.23, *p* < 0.001; Figure 3B). Planned comparison showed ARS effect (*p* < 0.001; Figure 3A). In females, we did not observe any PE or ARS effects (three-way repeated measures ANOVA: prenatal treatment, ARS, 2-min epoch with litter as a nested factor: main effect of epoch, *F*(8, 192) = 102.18, *p* < 0.001).

Lastly, we analyzed the AUC to examine habituation of locomotor activities under baseline condition and after ARS. In males, we did not observe an effect of PE. Only ARS effect was observed (two-way repeated measures ANOVA: prenatal treatment, ARS with litter as a nested factor: main effect of ARS, *F*(1, 10) = 27.05, *p* < 0.001; Figure 3C). We did not observe any PE or ARS effects in females.

### Prenatal ethanol exposure reduced the locomotor activities in the center zone under baseline condition in females, indicating increased anxiety-like behavior: Such an effect was observed in males only after ARS

**Figure 4:**
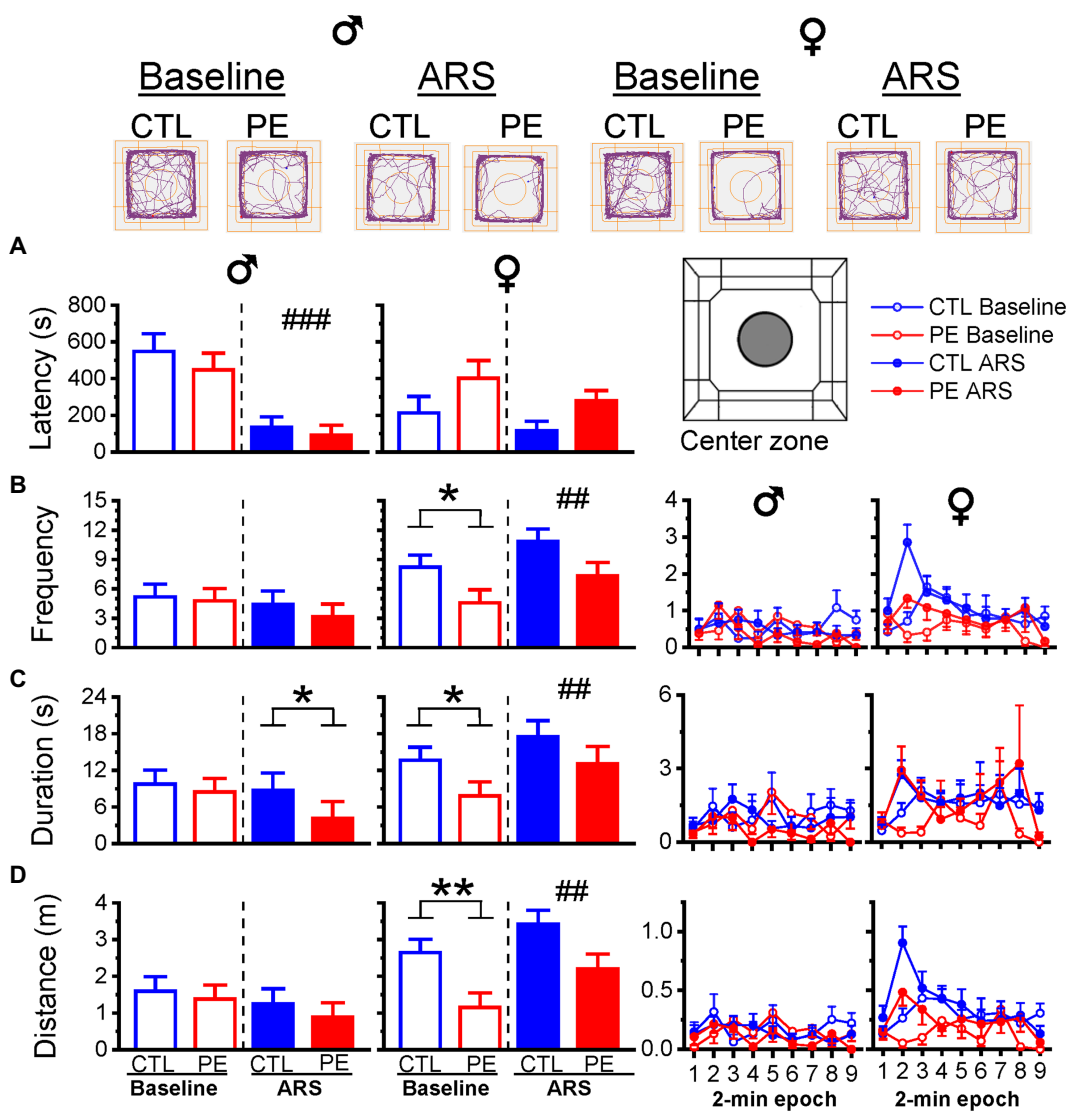
Prenatal ethanol exposure (PE) and acute restraint stress (ARS) effects on activities in the center zone. Upper panel reveals the representative tracking plots in the OF task. Prenatal ethanol exposure decreased the duration in the center zone after ARS in male rats **(C)**. Whereas, PE reduced the frequency, duration, and distance travelled under the baseline condition in female rats **(B–D)**. Prenatal ethanol did not impact the latency in males and females **(A)**. The right panels display the frequency, duration and distance of activities in the center zone in 2-min epoch. Data are represented as Mean ± SEM. *p ≤ 0.05, **p < 0.01, control vs. PE. ##*p* < 0.01, ###*p* < 0.001, baseline vs. ARS. CTL, control.

In males, prenatal treatment showed a decrease in duration in the center zone only after ARS, indicating increased anxiety-like behavior. Two-way repeated measures ANOVA with litter as a nested factor (prenatal treatment, ARS) showed a main effect of ARS in the latency of the first-time entry (*F*(1, 23) = 19.76, *p* < 0.001; Figure 4A). Planned comparisons after ANOVA showed a significant difference between control and PE males in duration after ARS (*p* = 0.05; Figure 4C). Prenatal ethanol exposure did not impact other center zone activities.

In females, PE led to reduced activities in the center zone under baseline condition but not after ARS (two-way repeated measures ANOVA with litter as a nested factor: prenatal treatment, ARS; latency of the first-time entry: main effect of prenatal treatment, *F*(1, 13) = 7.91, *p* < 0.05; Figure 4A; frequency: main effect of ARS, *F*(1, 13) = 12.18, *p* < 0.01; Figure 4B; duration: main effect of ARS, *F*(1, 13) = 10.92, *p* < 0.01; Figure 4C; distance: main effect of prenatal treatment, *F*(1, 13) = 6.73, *p* < 0.05, main effect of ARS, *F*(1, 13) = 10.31, *p* < 0.01; Figure 4D). Planned comparison after ANOVA showed differences between control and PE females under baseline condition (frequency, *p* < 0.05; duration, *p* = 0.05; distance, *p* < 0.01). Overall, center zone activities were relatively low over 18 min (Figures 4B–D).

Many OF studies use shorter duration of center zone activities in the OF task (e.g., 5 min) (Prut and Belzung, 2003) to infer anxiety-like behavior. We also analyzed the center zone activities in the first 6 min and did not find any PE or ARS effects in males. Prenatal ethanol exposure decreased the center zone distance but not other center zone measurements in females. Only ARS effect on center zone duration was observed (three-way repeated measures ANOVA with litter as a nested factor: prenatal treatment, ARS, 2-min epoch; duration: interaction effect of ARS and epoch, *F*(2, 48) = 5.82, *p* < 0.01; frequency: interaction effect of prenatal treatment and ARS, *F*(2, 48) = 3.41, *p* < 0.05; distance: interaction effect of ARS and epoch, *F*(2, 48) = 9.68, *p* < 0.001, main effect of prenatal treatment, *F*(1, 24) = 9.00, *p* < 0.01). Planned comparison showed ARS effect on duration (*p* < 0.05) and prenatal treatment effects on distance under basal condition (*p* < 0.01). Analyzing only 6 min of center zone activities is not sufficient to show group differences possibly due to very low level of activities.

### Prenatal ethanol exposure did not impact the durations in the edge, peripheral and corner zones

**Figure 5:**
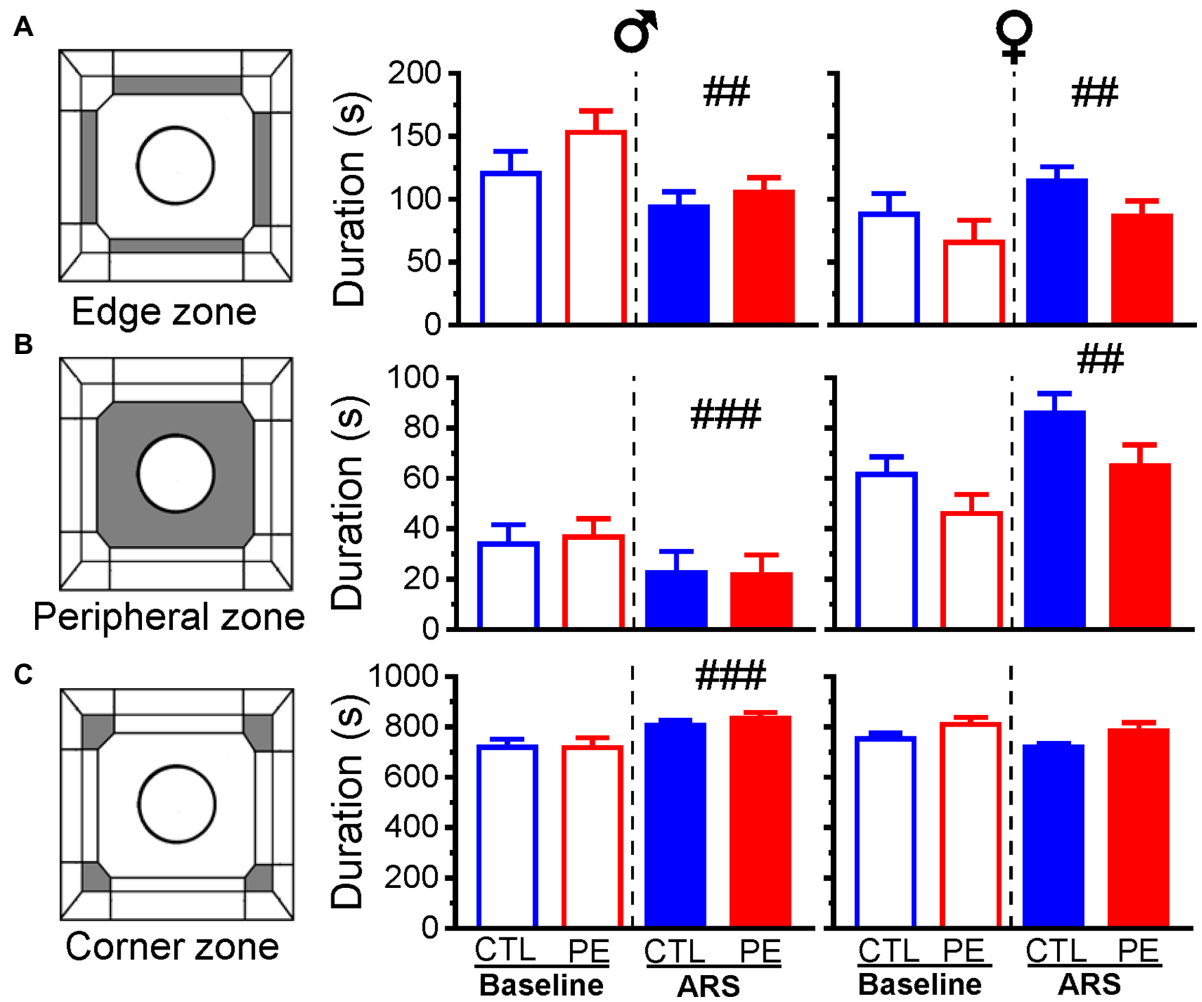
Effects of prenatal ethanol exposure (PE) and acute restraint stress (ARS) on duration spent in different zones in the OF: edge zone **(A)**, peripheral zone **(B)**, and corner zone **(C)** in the baseline and after ARS in both male and female rats. Prenatal ethanol exposure did not change the duration in any of the zones. Acute restraint stress exerted sex dimorphic effects on duration in each zone. Data are represented as Mean ± SEM. CTL, ##*p* < 0.01, ###*p* < 0.001, baseline condition vs. ARS.

We analyzed duration animal spent in the edge zone, the peripheral zone, and the corner zone in each sex using two-way repeated measures ANOVA with litter as a nested factor (prenatal treatment, ARS). We did not observe any PE effects. Nevertheless, ARS effects were observed in males and females, respectively. In males, main effects of ARS were found in the edge zone (*F*(1, 23) = 9.33, *p* < 0.01; Figure 5A), peripheral zone (*F*(1, 23) = 16.94, *p* < 0.001; Figure 5B) and corner zone (*F*(1, 23) = 19.66, *p* < 0.001; Figure 5C). In females, main effects of ARS were found in the edge zone (*F*(1, 24) = 8.36, *p* < 0.01) and peripheral zone (*F*(1, 24) = 11.49, *p* < 0.01).

### Prenatal ethanol exposure reduced rearing (vertical exploration) in the corner in both sexes: This effect was also observed in females after ARS

**Figure 6:**
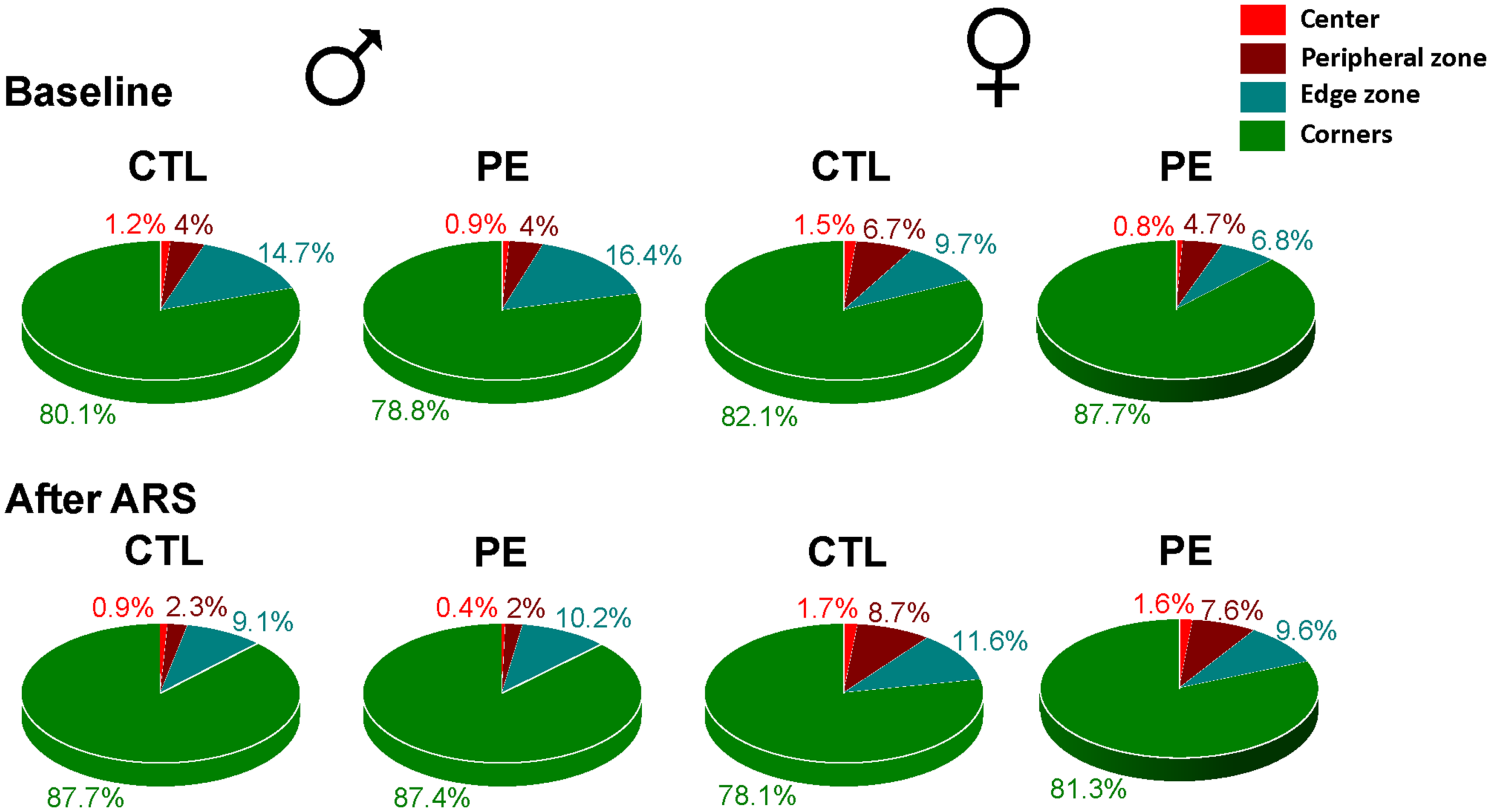
Percentages of time spent in the center, peripheral, edge, and corner zone in the OF task. Rats in all group spent >75% of time in the corner zone but <2% in the center zone.

**Figure 7:**
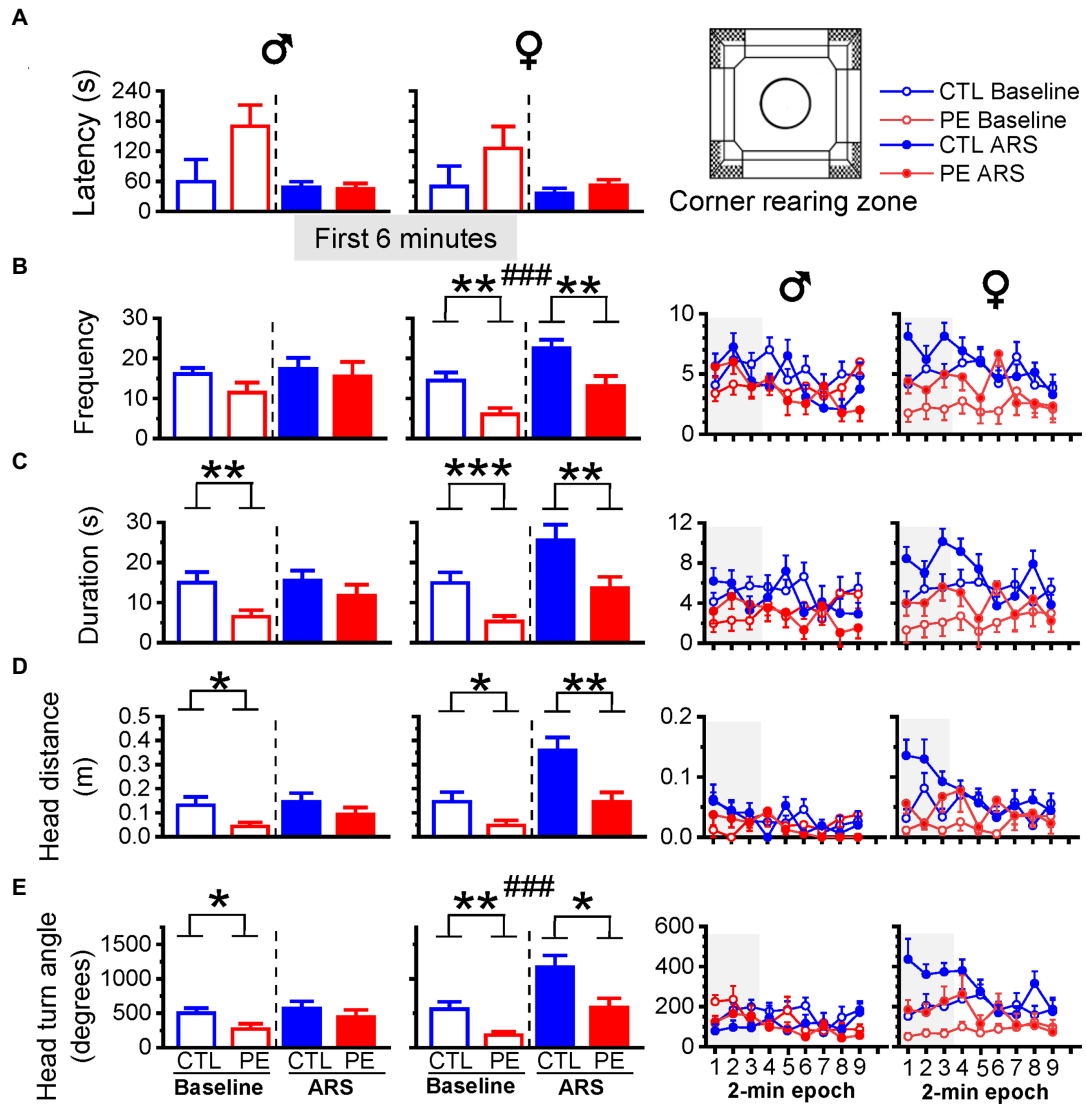
Effects of prenatal ethanol exposure (PE) and acute restraint stress (ARS) on rearing behaviors in the corner in the first 6 min of the OF task. In male rats, PE reduced the duration, head distance, head turn angle, indicating increased anxiety-like behavior **(C–E)**. In female rats, PE reduced the frequency, duration, head distance and the head turn angle under baseline condition and after ARS **(B–E)**. Prenatal ethanol did not impact the latency in males and females **(A)**. The right panels display the frequency, duration, head movement, and head turn angle of rearing in 2-min epoch. Data are represented as Mean ± SEM. *p ≤ 0.05, **p < 0.01, ***p < 0.001, control vs. PE. ###*p* < 0.001, baseline condition vs. ARS.

When we examined the duration spent in each zone (Figure 6), it was obvious that animals spent most of time in the corner zone (>75%) in contrast to the center zone (<2%). The major behavior animals engaged in the corner is rearing, which represents vertical exploratory activity. Increased rearing indicates lower anxiety (Zimcikova et al., 2017). We analyzed the latency of the first-time entry, frequency, duration, head distance, and head turn angle of rearing behavior (Figure 7). Movements such as head distance and head turn angle during rearing are important ethological indicators of exploratory behavior (Haddad et al., 2021).

We examined the first 6 min of rearing behavior because rats engaged in maximal rearing behavior in the beginning of the test. In males, PE decreased the duration, head distance, and head turn angle during rearing behavior in the corner indicating increased anxiety-like behavior (three-way repeated measures ANOVA with litter as a nested factor: prenatal treatment, ARS, 2-min epoch; duration: main effect of prenatal treatment, *F*(1, 23) = 6.15, *p* < 0.05; Figure 7C; head distance: main effect of prenatal treatment, *F*(1, 23) = 4.76, *p* < 0.05; Figure 7D; head turn angle: an interaction of prenatal treatment, ARS and epoch, *F*(2, 46) = 3.96, *p* < 0.05; Figure 7E). Planned comparisons after ANOVA confirmed that under basal condition, PE reduced rearing duration (*p* < 0.01), head distance (*p* < 0.05), and head turn angle (*p* < 0.05). The PE effects were not observed after ARS.

As for females, PE decreased rearing behavior in the frequency, duration, head distance, and head turn angle (three-way repeated measures ANOVA with litter as a nested factor: prenatal treatment, ARS, 2-min epoch; frequency: main effect of prenatal treatment, *F*(1, 24) = 12.96, *p* < 0.01, main effect of ARS, *F*(1, 24) = 22.42, *p* < 0.001; Figure 7B; duration: main effect of prenatal treatment, *F*(1, 24) = 10.00, *p* < 0.01, main effect of ARS, *F*(1, 24) = 18.05, *p* < 0.001; Figure 7C; head distance: an interaction of ARS and epoch, *F*(2, 48) = 3.21, *p* < 0.05; Figure 7D; head turn angle: main effect of prenatal treatment, *F*(1, 24) = 8.98, *p* < 0.01, main effect of ARS, *F*(1, 24) = 36.45, *p* < 0.001; Figure 7E). Planned comparisons showed that PE reduced rearing behaviors under baseline condition (frequency: *p* < 0.01; duration: *p* < 0.001; head distance: *p* < 0.05; head turn angle: *p* < 0.01) and after ARS (frequency: *p* < 0.01; duration: *p* < 0.01; head distance: *p* < 0.01; head turn angle: *p* < 0.05).

## Discussion

The current study reveals that heavy PE leads to increased anxiety-like behavior during adulthood in both sexes. The expression of anxiety-like behavior depends on ambient light levels. Group differences cannot be observed when ambient light is low (14 lux). At higher ambient light level, the anxiety-like behavior is observed only in PE females using the traditional index of reduced activities in the center zone of the OF. On the other hand, reduced activities in the center zone could be observed after acute stress in males. Importantly, we have observed that PE reduces vertical exploratory behavior (rearing) in the corner of the OF in both sexes under basal condition. Overall, PE does not impact locomotor activities or its habituation indicating the expression of anxiety-like behavior is not confounded by altered movement.

Animals tend to locomote and explore along the vertical surface in an unfamiliar, novel environment (Lever et al., 2006) instead of venturing into the center zone to avoid dangers (e.g., the threat from predators). Decreased activity level and duration in the center zone of the OF is commonly used to infer anxiety (Prut and Belzung, 2003). In this study, we first demonstrate that locomotor activity in the center zone is affected by ambient light level (Kuniishi et al., 2017). In lower ambient light setting (14 lux), animals show increased activities in the center zone compared to that in higher light setting (60 lux) and no PE effects are observed in either sex. In the higher light setting, we are able to observe that female PE rats show reduced locomotor activities in the center zone compared to controls, supporting PE leads to increased anxiety in females. The observations are consistent with those obtained from previous studies of early life stress showing the optimal light level for studying group differences in anxiety tasks is between 10–75 lux (Kuniishi et al., 2017). Ambient light level also impacts other anxiety tasks in a similar way such as the elevated-plus maze (Garcia et al., 2005). Taken together, sufficient ambient light is critical for the expression of anxiety-like behavior. Additional observation is that female rats have higher locomotor activities in general, which is also consistent with a previous study (Hellemans et al., 2010b). Prenatal ethanol exposure induced decreases in center zone activities is not observed in males. A clear decrease in center zone activity can be observed in male PE rats only when acute stress (ARS) was applied immediately before the OF test. The results indicate that PE effects on anxiety-like behavior interact with sex and acute stress.

We have observed that rats of both sexes spend very little time (<2%) in the center zone but more than 75% of the time in the corner zone of the OF. Therefore, we carefully examine the behavior in the corner zone and observe that animals often engage in rearing behavior, especially in the beginning of the OF test. Although rats also display rearing in the edge zone, its frequency is relatively low because rats spend limited time in this zone. When rats rear in the corner, they stand on the hind legs, extend their body upward, put their front paws on the wall, and engage in side-to-side head movement. Rearing is considered spontaneous vertical exploratory behavior to novelty in rodents which is similar to horizontal exploration. The rearing behavior observed is similar to that described for exploratory behavior in rodents for information gathering, food forage, or escape described in a previous publication (Lever et al., 2006). Rearing behavior had a negative correlation with anxiety level through confirmatory factor analysis (Brenes Saenz et al., 2006). The results from the present study show that duration of rearing and parameters of head movement during rearing are all decreased significantly in PE animals in both sexes. These observations support PE reduces exploratory behavior and increases anxiety. Overall, the PE-induced effect on rearing is not dependent on duration spent in the corner which are similar between control and PE rats. However, compared to males, females engage in more rearing behavior and in a more sustained pattern, consistent with greater locomotor activities in females. Taken together, we show that rearing behavior in the corner of the OF can be quantified in detail using a camera-based data acquisition system. Reduced rearing in the corner could be used as a reliable index of anxiety-like behavior in addition to activity/duration in the center zone in the OF task.

The current PE paradigm does not impact the overall locomotor activities of both sexes under higher ambient light. We use the traditional center zone activity as well a new measurement of corner rearing behavior to infer anxiety. Prenatal ethanol treatment does not cause the differences in center activities in male rats under basal condition. On the contrary, PE reduces the frequency, duration and distance of female rats in the center zone, indicating PE leads to increased anxiety-like behavior in female rats. On the other hand, PE reduces activities in the center zone (increases anxiety-like behavior) after acute stress in male but not female rats. Animals spend most of the time in the corner. When using reduced corner rearing behavior for increased anxiety, we observe increased anxiety in both male and female rats under the basal condition including reduced rearing duration, head distance and head turn angle. Increased anxiety-like behavior is only observed in females after acute stress. The above results show PE reduces activities in the center zone and rearing behavior. The results raise a possibility that these effects could be caused by impaired motor function in PE rats. However, observations from our present and previous studies do not support this notion. In the present study, there are no differences in overall locomotor activity in control and PE rats in either sex. In addition, when locomotor activity was evaluated in a confined space (42.5 × 22.5 × 19.25 cm) using the same PE paradigm in male rats in a previous study, we actually observed increased overall locomotor activity (Wang et al., 2019). In the same study, no group differences were found in rearing frequency in 60 min between control and PE rats (control: 163.08 ± 14.68 vs. PE: 182.58 ± 15.19; unpublished data).Anxiety behavior is also affected by acute stress (Sturman et al., 2018). Strong evidence shows that PE leads to augmented stress reactivity indicated by increased cortisol level (Hellemans et al., 2010a,b). In the present study, we use 2 h restraint right before the OF test to examine effects of acute stress on anxiety behavior. The results show acute stress increases anxiety-like behavior indicated by decreased activities in center zone in males which is not observed under basal conditions. Acute stress does not further augment anxiety-like behavior in females. These observations are consistent with previous studies showing ARS increases stress reactivity (Sturman et al., 2018). When corner rearing behavior is evaluated, we have not observed acute stress further increases anxiety-like behavior in either males or females. However, we observe an overall increase in rearing activities in females after acute stress. Taken together, PE effects on rearing behavior are not altered by acute stress.

It has been found that PE impacts anxiety-like behavior. However, the PE effects on anxiety-like behavior are inconsistent due to different behavioral tasks, ethanol exposure paradigms, sex and age. Acute ethanol exposure on GD 12 *via* intraperitoneal injection reduces social preference (a measurement of social anxiety-like behavior) in female adult rats but not in males (Diaz et al., 2020). Ethanol vapor exposure for 6 h on GD 12 enhances anxiety-like behavior in male adolescent rats in the novelty-induced hypophagia task, light–dark box, and OF, but not in female adolescent offspring (Rouzer et al., 2017). Ethanol exposure (4 g/kg) on GD 16 and 17 *via* intraperitoneal injection increases anxiety in adolescent male and female mice (Hwang and Hashimoto-Torii, 2022) in the elevated plus-maze task. Chronic ethanol exposure with liquid diet during GDs 10–21 leads to reduced anxiety-like behavior in adult male rats in the elevated plus-maze task (Ohta et al., 2010). Conversely, chronic ethanol exposure from GDs 7–20 using intragastric intubation leads to increased anxiety-like behavior in male offspring (Dursun et al., 2006). Another study shows no impact of chronic ethanol exposure using ethanol vapor during GDs 5–20 in rats of both sexes using an OF task (Breit et al., 2022). Using the current chronic heavy PE paradigm, we observed increased anxiety-like behavior using elevated plus-maze and zero maze tasks in adult male rats (Oubraim et al., 2022), which is consistent with the current study. The inconsistent observations of anxiety-like behavior after chronic PE could be due to PE paradigms as well as different behavioral tasks used. Some researchers resort to more than one behavioral tasks to measure anxiety-like behavior (Cullen et al., 2013; Wieczorek et al., 2015; Rouzer et al., 2017) in order to reach a clear conclusion. Results from the present study indicate rearing behavior in the corner of the OF task is a reliable measurement for anxiety-like behavior in both sexes. Rearing behavior in the corner zone is also multifaceted and complex. Behavioral parameters such as head movement and head turn angle, which are specific features of vertical exploratory behavior, can provide additional ethological information. Combined analyses of horizontal and vertical exploratory behavior in the OF task could provide a reasonable and simple approach to investigate anxiety-like behavior.

The mechanism of anxiety is complex and often interacts with depression-like behaviors (Hellemans et al., 2010b). In clinic studies, Niclasen et al. (2014) observed prenatal exposure to binge drinking in pregnancy was associated positively with impaired behavioral and emotional development in preadolescent boys. In adults with FASD, females show much higher propensity for depression and anxiety than males (Famy et al., 1998). Together, strong evidence showed that PE increases anxiety-like behavior. Therefore, it is crucial to understand the underlying neuronal mechanisms caused by PE in order to develop effective intervention strategies.

The neural circuitries underlying rearing are not fully understood. However, it has been proposed that the hippocampus is involved (Sturman et al., 2018). When animals rear, better view is obtained for enhanced space exploration (Lever et al., 2006). The hippocampus is involved in space navigation and interconnected with stress centers including hypothalamus, amygdala, and other limbic structures (Bannerman et al., 2014). Specifically, the ventral hippocampus interconnection with the hypothalamus plays an important role in regulating stress responses (Tannenholz et al., 2014). Prenatal alcohol exposure is known to affect hippocampal functions (Sanchez et al., 2019; Dodge et al., 2020) and alter the sensitivity of HPA axis (Hellemans et al., 2010b; Wieczorek et al., 2015) which could mediate increased anxiety-like behavior. Other neural circuitries and neurotransmitter systems could also mediate PE-induced reduction in rearing/increased anxiety. Studies have reported that PE persistently alters several neurotransmitter systems involved in regulating emotional behavior, including the serotonin (5-HT) system. Prenatal ethanol exposure causes lower density of 5-HT neurons (Druse et al., 1991; Ohta et al., 2010), fewer 5-HT fibers, impaired 5-HT projections (Zhou et al., 2001), which suggest PE leads to expression of the anxiety-like behavior in animals. A recent study has discovered that PE induced persistent activation of 5-HT neurons and its glutamate synapses by increasing nitrergic function and degrading endocannabinoids signaling in the dorsal raphe 5-HT neurons (Oubraim et al., 2022). This activation exertes the anxiogenic effect, thereby PE animals express anxiety-like behavior. Through the morphological studies, prenatal ethanol exposure, regardless of the consumption volume, results in neuronal morphological alterations in the amygdala (Cullen et al., 2013) and striatum (Ma, 2019). These changes could mediate increased anxiety-like behavior.

## Conclusion

The results from the present study show chronic heavy PE causes a persistent increase in anxiety-like behavior in adulthood in both sexes. This conclusion is based on improvement of traditional OF task. Specifically, the vertical exploratory behavior (rearing) in the corner zone is analyzed in detail, which has not been emphasized before. This approach can be a reliable indicator for anxiety-like behavior in general.

## Data availability statement

The raw data supporting the conclusions of this article will be made available by the authors, without undue reservation.

## Ethics statement

The animal study was reviewed and approved by University at Buffalo, Institutional Animal Care and Use Committee.

## Author contributions

VM and FK performed behavioral tests. A-LW, VM, RW, KH, and R-YS completed data analyses. KH, SO, and R-YS conducted breeding and prenatal ethanol treatments. R-YS, VM, KH, RW, and SH-D participated in the experimental design. R-YS and SH-D directed the project. A-LW and R-YS wrote the manuscript. All authors contributed to the article and approved the submitted version.

## Funding

This study was support by grant AA026421 and AA026601 from the National Institutes of Health awarded to R-YS and SH-D.

## Conflict of interest

The authors declare that the research was conducted in the absence of any commercial or financial relationships that could be construed as a potential conflict of interest.

## Publisher’s note

All claims expressed in this article are solely those of the authors and do not necessarily represent those of their affiliated organizations, or those of the publisher, the editors and the reviewers. Any product that may be evaluated in this article, or claim that may be made by its manufacturer, is not guaranteed or endorsed by the publisher.

## References

[ref1] Aghaie C. I. Hausknecht K. A. Wang R. Dezfuli P. H. Haj-Dahmane S. Kane C. J. M.. (2020). Prenatal ethanol exposure and postnatal environmental intervention Alter dopaminergic neuron and microglia morphology in the ventral tegmental area during adulthood. Alcohol. Clin. Exp. Res. 44, 435–444. doi: 10.1111/acer.14275, PMID: 31872887 PMC7153307

[ref2] Ba A. Seri B. V. Han S. H. (1996). Thiamine administration during chronic alcohol intake in pregnant and lactating rats: effects on the offspring neurobehavioural development. Alcohol Alcohol. 31, 27–40. doi: 10.1093/oxfordjournals.alcalc.a008113, PMID: 8672172

[ref3] Bannerman D. M. Sprengel R. Sanderson D. J. McHugh S. B. Rawlins J. N. Monyer H.. (2014). Hippocampal synaptic plasticity, spatial memory and anxiety. Nat. Rev. Neurosci. 15, 181–192. doi: 10.1038/nrn3677 24552786

[ref4] Bartolomucci A. Gioiosa L. Chirieleison A. Ceresini G. Parmigiani S. Palanza P. (2004). Cross fostering in mice: behavioral and physiological carry-over effects in adulthood. Genes Brain Behav. 3, 115–122. doi: 10.1111/j.1601-183x.2003.00059.x, PMID: 15005720

[ref5] Breit K. R. Rodriguez C. G. Lei A. Hussain S. Thomas J. D. (2022). Effects of prenatal alcohol and delta-9-tetrahydrocannabinol exposure via electronic cigarettes on motor development. Alcohol. Clin. Exp. Res. 46, 1408–1422. doi: 10.1111/acer.14892, PMID: 35722858 PMC9427686

[ref6] Brenes Saenz J. C. Villagra O. R. Fornaguera Trias J. (2006). Factor analysis of forced swimming test, sucrose preference test and open field test on enriched, social and isolated reared rats. Behav. Brain Res. 169, 57–65. doi: 10.1016/j.bbr.2005.12.001, PMID: 16414129

[ref7] Briski K. Gillen E. (2001). Differential distribution of Fos expression within the male rat preoptic area and hypothalamus in response to physical vs. psychological stress. Brain Res. Bull. 55, 401–408. doi: 10.1016/s0361-9230(01)00532-9, PMID: 11489348

[ref8] Carobrez A. P. Bertoglio L. J. (2005). Ethological and temporal analyses of anxiety-like behavior: the elevated plus-maze model 20 years on. Neurosci. Biobehav. Rev. 29, 1193–1205. doi: 10.1016/j.neubiorev.2005.04.017, PMID: 16084592

[ref9] Cullen C. L. Burne T. H. Lavidis N. A. Moritz K. M. (2013). Low dose prenatal ethanol exposure induces anxiety-like behaviour and alters dendritic morphology in the basolateral amygdala of rat offspring. PLoS One 8:e54924. doi: 10.1371/journal.pone.0054924, PMID: 23383000 PMC3559882

[ref10] Diaz M. R. Johnson J. M. Varlinskaya E. I. (2020). Increased ethanol intake is associated with social anxiety in offspring exposed to ethanol on gestational day 12. Behav. Brain Res. 393:112766. doi: 10.1016/j.bbr.2020.112766, PMID: 32535179

[ref11] Diaz M. R. Mooney S. M. Varlinskaya E. I. (2016). Acute prenatal exposure to ethanol on gestational day 12 elicits opposing deficits in social behaviors and anxiety-like behaviors in Sprague Dawley rats. Behav. Brain Res. 310, 11–19. doi: 10.1016/j.bbr.2016.05.003, PMID: 27154534 PMC4893948

[ref12] Dodge N. C. Thomas K. G. F. Meintjes E. M. Molteno C. D. Jacobson J. L. Jacobson S. W. (2020). Reduced hippocampal volumes partially mediate effects of prenatal alcohol exposure on spatial navigation on a virtual water maze task in children. Alcohol. Clin. Exp. Res. 44, 844–855. doi: 10.1111/acer.14310, PMID: 32196695 PMC7166198

[ref13] Druse M. J. Kuo A. Tajuddin N. (1991). Effects of in utero ethanol exposure on the developing serotonergic system. Alcohol. Clin. Exp. Res. 15, 678–684. doi: 10.1111/j.1530-0277.1991.tb00578.x, PMID: 1928643

[ref14] Dursun I. Jakubowska-Dogru E. Uzbay T. (2006). Effects of prenatal exposure to alcohol on activity, anxiety, motor coordination, and memory in young adult Wistar rats. Pharmacol. Biochem. Behav. 85, 345–355. doi: 10.1016/j.pbb.2006.09.001, PMID: 17049371

[ref15] Famy C. Streissguth A. P. Unis A. S. (1998). Mental illness in adults with fetal alcohol syndrome or fetal alcohol effects. Am. J. Psychiatry 155, 552–554. doi: 10.1176/ajp.155.4.552 9546004

[ref16] Fryer S. L. McGee C. L. Matt G. E. Riley E. P. Mattson S. N. (2007). Evaluation of psychopathological conditions in children with heavy prenatal alcohol exposure. Pediatrics 119, e733–e741. doi: 10.1542/peds.2006-1606, PMID: 17332190

[ref17] Garcia A. M. Cardenas F. P. Morato S. (2005). Effect of different illumination levels on rat behavior in the elevated plus-maze. Physiol. Behav. 85, 265–270. doi: 10.1016/j.physbeh.2005.04.007 15927214

[ref18] Giberson P. K. Weinberg J. (1997). Effect of surrogate fostering on splenic lymphocytes in fetal ethanol exposed rats. Alcohol. Clin. Exp. Res. 21, 44–55. doi: 10.1111/j.1530-0277.1997.tb03727.x, PMID: 9046372

[ref19] Greenmyer J. R. Klug M. G. Kambeitz C. Popova S. Burd L. (2018). A multicountry updated assessment of the economic impact of fetal alcohol Spectrum disorder: costs for children and adults. J. Addict. Med. 12, 466–473. doi: 10.1097/ADM.0000000000000438, PMID: 30383615

[ref20] Haddad F. L. Ghahremani M. de Oliveira C. Doornaert E. E. Johnston K. D. Everling S.. (2021). A novel three-choice touchscreen task to examine spatial attention and orienting responses in rodents. eNeuro 8, ENEURO.0032–ENEU20.2021. doi: 10.1523/ENEURO.0032-20.2021, PMID: 33789926 PMC8272401

[ref21] Hellemans K. G. Sliwowska J. H. Verma P. Weinberg J. (2010a). Prenatal alcohol exposure: fetal programming and later life vulnerability to stress, depression and anxiety disorders. Neurosci. Biobehav. Rev. 34, 791–807. doi: 10.1016/j.neubiorev.2009.06.004, PMID: 19545588 PMC5518679

[ref22] Hellemans K. G. Verma P. Yoon E. Yu W. K. Young A. H. Weinberg J. (2010b). Prenatal alcohol exposure and chronic mild stress differentially alter depressive- and anxiety-like behaviors in male and female offspring. Alcohol. Clin. Exp. Res. 34, 633–645. doi: 10.1111/j.1530-0277.2009.01132.x, PMID: 20102562 PMC4833468

[ref23] Hwang H. M. Hashimoto-Torii K. (2022). Activation of the anterior cingulate cortex ameliorates anxiety in a preclinical model of fetal alcohol spectrum disorders. Transl. Psychiatry 12:24. doi: 10.1038/s41398-022-01789-1, PMID: 35058425 PMC8776849

[ref24] Keiver K. Bertram C. P. Orr A. P. Clarren S. (2015). Salivary cortisol levels are elevated in the afternoon and at bedtime in children with prenatal alcohol exposure. Alcohol 49, 79–87. doi: 10.1016/j.alcohol.2014.11.004, PMID: 25583378

[ref25] Kingdon D. Cardoso C. McGrath J. J. (2016). Research review: executive function deficits in fetal alcohol spectrum disorders and attention-deficit/hyperactivity disorder – a meta-analysis. J. Child Psychol. Psychiatry 57, 116–131. doi: 10.1111/jcpp.12451, PMID: 26251262 PMC5760222

[ref26] Kuniishi H. Ichisaka S. Yamamoto M. Ikubo N. Matsuda S. Futora E.. (2017). Early deprivation increases high-leaning behavior, a novel anxiety-like behavior, in the open field test in rats. Neurosci. Res. 123, 27–35. doi: 10.1016/j.neures.2017.04.012, PMID: 28450152

[ref27] Lange S. Probst C. Gmel G. Rehm J. Burd L. Popova S. (2017). Global prevalence of fetal alcohol Spectrum disorder among children and youth: a systematic review and meta-analysis. JAMA Pediatr. 171, 948–956. doi: 10.1001/jamapediatrics.2017.1919, PMID: 28828483 PMC5710622

[ref28] Lazic S. E. Essioux L. (2013). Improving basic and translational science by accounting for litter-to-litter variation in animal models. BMC Neurosci. 14:37. doi: 10.1186/1471-2202-14-37, PMID: 23522086 PMC3661356

[ref29] Lever C. Burton S. O'Keefe J. (2006). Rearing on hind legs, environmental novelty, and the hippocampal formation. Rev. Neurosci. 17, 111–133. doi: 10.1515/revneuro.2006.17.1-2.111, PMID: 16703946

[ref30] Ma Y. Y. (2019). Striatal morphological and functional alterations induced by prenatal alcohol exposure. Pharmacol. Res. 142, 262–266. doi: 10.1016/j.phrs.2019.02.022, PMID: 30807864 PMC7814345

[ref31] Mattson S. N. Crocker N. Nguyen T. T. (2011). Fetal alcohol spectrum disorders: neuropsychological and behavioral features. Neuropsychol. Rev. 21, 81–101. doi: 10.1007/s11065-011-9167-9, PMID: 21503685 PMC3410672

[ref32] May P. A. Baete A. Russo J. Elliott A. J. Blankenship J. Kalberg W. O.. (2014). Prevalence and characteristics of fetal alcohol spectrum disorders. Pediatrics 134, 855–866. doi: 10.1542/peds.2013-3319, PMID: 25349310 PMC4210790

[ref33] May P. A. Gossage J. P. Kalberg W. O. Robinson L. K. Buckley D. Manning M.. (2009). Prevalence and epidemiologic characteristics of FASD from various research methods with an emphasis on recent in-school studies. Dev. Disabil. Res. Rev. 15, 176–192. doi: 10.1002/ddrr.68, PMID: 19731384

[ref34] Niclasen J. Nybo Andersen A. M. Teasdale T. W. Strandberg-Larsen K. (2014). Prenatal exposure to alcohol, and gender differences on child mental health at age seven years. J. Epidemiol. Community Health 68, 224–232. doi: 10.1136/jech-2013-202956, PMID: 24218073

[ref35] Ohta K. Sakata-Haga H. Fukui Y. (2010). Alteration in anxiety-related behaviors and reduction of serotonergic neurons in raphe nuclei in adult rats prenatally exposed to ethanol. Congenit Anom (Kyoto) 50, 105–114. doi: 10.1111/j.1741-4520.2010.00269.x, PMID: 20156240

[ref36] Osborn J. A. Kim C. K. Steiger J. Weinberg J. (1998). Prenatal ethanol exposure differentially alters behavior in males and females on the elevated plus maze. Alcohol. Clin. Exp. Res. 22, 685–696. doi: 10.1111/j.1530-0277.1998.tb04312.x, PMID: 9622451

[ref37] Oubraim S. Wang R. Hausknecht K. Kaczocha M. Shen R. Y. Haj-Dahmane S. (2022). Prenatal ethanol exposure causes anxiety-like phenotype and alters synaptic nitric oxide and endocannabinoid signaling in dorsal raphe nucleus of adult male rats. Transl. Psychiatry 12:440. doi: 10.1038/s41398-022-02210-7, PMID: 36216807 PMC9550821

[ref38] Prut L. Belzung C. (2003). The open field as a paradigm to measure the effects of drugs on anxiety-like behaviors: a review. Eur. J. Pharmacol. 463, 3–33. doi: 10.1016/s0014-2999(03)01272-x, PMID: 12600700

[ref39] Pyman P. Collins S. E. Muggli E. Testa R. Anderson P. J. (2021). Cognitive and behavioural attention in children with low-moderate and heavy doses of prenatal alcohol exposure: a systematic review and meta-analysis. Neuropsychol. Rev. 31, 610–627. doi: 10.1007/s11065-021-09490-8, PMID: 33656703

[ref40] Rouzer S. K. Cole J. M. Johnson J. M. Varlinskaya E. I. Diaz M. R. (2017). Moderate maternal alcohol exposure on gestational day 12 impacts anxiety-like behavior in offspring. Front. Behav. Neurosci. 11:183. doi: 10.3389/fnbeh.2017.00183, PMID: 29033803 PMC5626811

[ref41] Sanchez L. M. Goss J. Wagner J. Davies S. Savage D. D. Hamilton D. A.. (2019). Moderate prenatal alcohol exposure impairs performance by adult male rats in an object-place paired-associate task. Behav. Brain Res. 360, 228–234. doi: 10.1016/j.bbr.2018.12.014, PMID: 30529401 PMC6324964

[ref42] Shen R. Y. Hannigan J. H. Kapatos G. (1999). Prenatal ethanol reduces the activity of adult midbrain dopamine neurons. Alcohol. Clin. Exp. Res. 23, 1801–1807. doi: 10.1111/j.1530-0277.1999.tb04076.x, PMID: 10591597

[ref43] Sturman O. Germain P. L. Bohacek J. (2018). Exploratory rearing: a context- and stress-sensitive behavior recorded in the open-field test. Stress 21, 443–452. doi: 10.1080/10253890.2018.1438405, PMID: 29451062

[ref44] Tang S. Xu S. Waddell J. Zhu W. Gullapalli R. P. Mooney S. M. (2019). Functional connectivity and metabolic alterations in medial prefrontal cortex in a rat model of fetal alcohol Spectrum disorder: a resting-state functional magnetic resonance imaging and in vivo proton magnetic resonance spectroscopy study. Dev. Neurosci. 41, 67–78. doi: 10.1159/000499183, PMID: 30999297 PMC7424565

[ref45] Tannenholz L. Jimenez J. C. Kheirbek M. A. (2014). Local and regional heterogeneity underlying hippocampal modulation of cognition and mood. Front. Behav. Neurosci. 8:147. doi: 10.3389/fnbeh.2014.00147, PMID: 24834033 PMC4018538

[ref46] Wang R. Hausknecht K. A. Haj-Dahmane S. Shen R. Y. Richards J. B. (2018a). Decreased environmental complexity during development impairs habituation of reinforcer effectiveness of sensory stimuli. Behav. Brain Res. 337, 53–60. doi: 10.1016/j.bbr.2017.09.032, PMID: 28943426 PMC5676459

[ref47] Wang R. Hausknecht K. A. Shen Y. L. Haj-Dahmane S. Vezina P. Shen R. Y. (2018b). Environmental enrichment reverses increased addiction risk caused by prenatal ethanol exposure. Drug Alcohol Depend. 191, 343–347. doi: 10.1016/j.drugalcdep.2018.07.013, PMID: 30176547 PMC6178821

[ref48] Wang R. Martin C. D. Lei A. L. Hausknecht K. A. Ishiwari K. Oubraim S.. (2021). Moderate prenatal ethanol exposure leads to attention deficits in both male and female rats. Alcohol. Clin. Exp. Res. 45, 1122–1135. doi: 10.1111/acer.14599, PMID: 33730380 PMC11774337

[ref49] Wang R. Martin C. D. Lei A. L. Hausknecht K. A. Ishiwari K. Richards J. B.. (2020). Prenatal ethanol exposure leads to attention deficits in both male and female rats. Front. Neurosci. 14:12. doi: 10.3389/fnins.2020.00012, PMID: 32038156 PMC6992663

[ref50] Wang R. Martin C. D. Lei A. L. Hausknecht K. A. Turk M. Micov V.. (2022). Prenatal ethanol exposure impairs sensory processing and habituation to visual stimuli, effects normalized by enrichment of postnatal environmental. Alcohol. Clin. Exp. Res. 46, 891–906. doi: 10.1111/acer.14818, PMID: 35347730 PMC9122102

[ref51] Wang R. Shen Y. L. Hausknecht K. A. Chang L. Haj-Dahmane S. Vezina P.. (2019). Prenatal ethanol exposure increases risk of psychostimulant addiction. Behav. Brain Res. 356, 51–61. doi: 10.1016/j.bbr.2018.07.030, PMID: 30076855 PMC7395677

[ref52] Wieczorek L. Fish E. W. O'Leary-Moore S. K. Parnell S. E. Sulik K. K. (2015). Hypothalamic-pituitary-adrenal axis and behavioral dysfunction following early binge-like prenatal alcohol exposure in mice. Alcohol 49, 207–217. doi: 10.1016/j.alcohol.2015.01.005, PMID: 25709101 PMC4414725

[ref53] Zhou F. C. Sari Y. Zhang J. K. Goodlett C. R. Li T. (2001). Prenatal alcohol exposure retards the migration and development of serotonin neurons in fetal C57BL mice. Brain Res. Dev. Brain Res. 126, 147–155. doi: 10.1016/s0165-3806(00)00144-9, PMID: 11248348

[ref54] Zimcikova E. Simko J. Karesova I. Kremlacek J. Malakova J. (2017). Behavioral effects of antiepileptic drugs in rats: are the effects on mood and behavior detectable in open-field test? Seizure 52, 35–40. doi: 10.1016/j.seizure.2017.09.015, PMID: 28957723

